# Validation of the Italian version of the Eating-Related Eco-Concern Questionnaire: insights into its relationship with orthorexia nervosa

**DOI:** 10.3389/fpsyg.2024.1441561

**Published:** 2024-11-27

**Authors:** Lucia Tecuta, Giulia Casu, Elena Tomba

**Affiliations:** Department of Psychology, University of Bologna, Bologna, Italy

**Keywords:** eco-concerns, eco-distress, dietary behaviors, eating disorders, orthorexia nervosa, validation, psychometrics

## Abstract

**Introduction:**

Urgent calls for research on the relationship between climate change concerns and eating disorder risk have been made. This study aimed to validate an Italian version of the Eating-Related Eco-Concern Questionnaire (EREC), a brief unidimensional measure of eating behaviors related to eco-concern.

**Methods:**

Six hundred and sixty-three adults (85% females, mean age 37 ± 12 years) completed the EREC, Climate Change Worry Scale (CCWS), Eating Disorder Examination-Questionnaire (EDE-Q), Eating Habits Questionnaire for orthorexia nervosa symptoms (EHQ-21), and questions on dietary habits and motivations, and past experiences of extreme climate events.

**Results:**

Confirmatory factor analysis results indicated that the original one-factor model showed acceptable fit to the data after including the error covariation between two pairs of items. Internal consistency was adequate, and EREC scores correlated positively and strongly with CCWS scores. Participants scored significantly lower in EREC than in CCWS, indicating greater climate-related concerns in general terms than relating specifically to eating. While EREC was unrelated to EDE-Q scores, weak-to-moderate correlations were observed with EHQ-21 subscale and total scores. Pro-environmental and/or ethical reasons for current diet and personal experience of extreme climate events were associated with significantly higher EREC scores.

**Discussion:**

The Italian EREC appears to be a valid and reliable tool for the screening of eating-related concerns and behaviors related to climate change. Ecological concerns may represent a healthy adaptive response, but the EREC can serve as a valuable tool to identify individuals whose eating behaviors related to eco-concern might warrant further clinical attention due to potential risks of developing rigid or unhealthy patterns.

## 1 Introduction

Climate change, characterized by long-term shifts in weather patterns and temperatures stemming from human activities, has recently intensified. Its far-reaching consequences encompass not only damaging effects to food security and living conditions across the globe, but profound implications for human health (Filippini et al., 2024) as well as for mental health (IPCC, 2022; Charlson et al., 2021) with a notable percentage of individuals exposed to extreme climate events exhibiting subsequent psychiatric conditions (Filippini et al., 2024).

The study of the psychological effects of climate change has yielded the term “eco-distress” to capture the emotional distress specifically linked to the ecological and climate crisis, encompassing feelings of anxiety, helplessness, guilt, and despair that constitutes an important risk to mental health (Agoston et al., 2022). In particular, eco-anxiety or climate anxiety (Coffey et al., 2021; Hogg et al., 2021; Hickman et al., 2021), somewhat used interchangeably albeit with nuanced differences (Pihkala, 2019, 2020) has been found to range from appropriate, rational and adaptive with associations with pro-environmental behaviors on the one hand (Verplanken et al., 2020; Jain and Jain, 2022; Heeren et al., 2023; Rocchi et al., 2023), to pathological on the other, in extreme cases being associated with worse mental health outcomes in terms of anxiety, depression, and suicidality (Ellis and Albrecht, 2017; Helm et al., 2018; Heinzel et al., 2023). Moreover, it is prevalent among the youth (Hickman et al., 2021) who report greater levels of climate and eco-anxiety compared to older individuals (Clayton and Karazsia, 2020; Rocchi et al., 2023).

Nonetheless, while previous studies have established connections between various conceptualizations of eco-distress, specifically eco-anxiety or climate anxiety, and psychopathology, little attention has been directed toward its potential correlation with altered eating patterns, referring to changes in typical patterns of eating, such as consuming too much or too little food, avoiding certain food groups, or engaging in behaviors like binge eating, purging, or extreme dieting, and eating disorder (ED) risk. Indeed, urgent calls for further research into this area have been made. Rodgers et al. (2023) have argued that climate change might be correlated with increased risk for EDs, an exacerbation in symptoms, or poor clinical outcomes through various pathways. Clinical observations have highlighted the possibility that some individuals might reduce their food intake and eliminate food groups altogether in an effort to reduce their carbon footprint due to environmental concerns and ethical considerations regarding their contribution to climate change. The creation of the Eating-Related Eco-Concern Questionnaire by Qi et al. (2022) has offered a fundamental first step in unveiling the relationship between eco-concern and altered eating behaviors, positing a plausible association between the two phenomena. Qi et al. (2022) and Rodgers et al. (2023) argue that concerns about the ecological and climate crisis, marked by distress stemming from the environmental crisis, may interact with disordered eating manifestations such as dietary restraint and weight concerns given the impact on mental health.

The current study aims to explore the psychometric properties of the Eating-Related Eco-Concern (EREC) scale (Qi et al., 2022) in the Italian context. Concerns about climate change are increasingly relevant in Italy, an area particularly sensitive geographically speaking to the effects of climate change and associated adverse health effects (IPCC, 2022). According to a survey conducted by King’s College London, a significant majority of Italians, approximately 87%, express profound concern regarding the implications of climate change. Among them, 54% perceive climate change as actively detrimental to their nation presently with an additional 16% foreseeing adverse effects within the coming decade (King’s College of London, 2022). Moreover, while prevalence rates of EDs in Mediterranean countries such as Italy do not seem to significantly differ from those of non-Mediterranean countries, greater eating disorder-related attitudes have been reported in Italian adolescents compared to Anglo-Saxon counterparts (Santonastaso et al., 1996).

Within the context of ED risk and the ecological and climate crisis, it is increasingly relevant to consider the rise of vegetarianism in response to the climate crisis (Mayrhofer et al., 2024), and how this may imply greater health risks as research suggests greater risk in developing orthorexia nervosa in lacto-vegetarians (Dittfeld et al., 2017; Parra-Fernández et al., 2020). Orthorexia nervosa, a proposed eating disorder (Hyrnik et al., 2016), is characterized by an obsessive preoccupation with consuming healthy or “pure” foods to the detriment of overall well-being and might correlate with eco-anxiety about food choices. Individuals with eco-anxiety might become excessively worried about consuming foods whose quality is affected by climate change (Tchonkouang et al., 2024) and that might be detrimental for themselves, leading to heightened stress, guilt, or obsession surrounding food selection and consumption.

The study aims to explore the psychometric properties of the Italian version of the EREC scale by assessing its internal structure, internal consistency reliability, and relations with other variables. It was hypothesized that the EREC would demonstrate adequate reliability and validity. For evidence of validity based on internal structure, it was expected that the one-factor model identified in the original EREC validation study (Qi et al., 2022) would be confirmed in the Italian version. For evidence of validity based on relation with other variables, it was anticipated that EREC scores would correlate positively and strongly with climate change worry, positively and weakly with disordered eating, and that lower scores would be reported for eating behaviors related to eco-concern compared to climate change worry, as observed in the original validation study (Qi et al., 2022). Given the conceptual overlaps regarding similar food choices (i.e., avoidance of highly processed foods and animal-based products) and food-related concerns (Dunn and Bratman, 2016), associations with orthorexia nervosa were also considered, with expectations of positive correlations. Finally, based on the literature, negative correlations were hypothesized with age (Clayton and Karazsia, 2020; Rocchi et al., 2023) and body mass index (BMI) (given the restrictive nature of EREC items), while higher EREC scores were expected for individuals whose dietary choices are motivated by environmental concerns and ethical considerations (Chung et al., 2023; Heeren et al., 2023), as well as for those who have experienced climate change events (Reser and Bradley, 2020; Clayton, 2020).

## 2 Methods

### 2.1 Procedure

The study recruited general population participants online via a QR code that led to a Qualtrics page. The QR code was shared on major social media and social networks, along with a snowballing approach among researchers’ contacts. IP addresses were not collected to ensure anonymity. Inclusion criteria was being over 18 years old and fluent in Italian. Participation was voluntary and could be canceled at any time without provision of reasons or negative consequences. No compensation was offered for participation. The study was approved by the Bioethics Committee of the University of Bologna (approval no. 0155312 on 06/08/2023), and all participants provided informed consent.

### 2.2 Measures

An *ad hoc* survey to collect socio-demographic information including age, gender, nationality, educational level (highest degree obtained), current job status (unemployed/employed), current income level (difficulty in making ends meet), body mass index (BMI), and dietary habits was administered. The survey also included *ad hoc* closed-ended (yes/no) questions regarding motivations for dietary choices (health reasons, weight management, ethical/moral reasons, environmental concerns, taste preferences, medical reasons, and religious/spiritual values) and whether the respondent had ever experienced a climate-related event including hailstorms, extreme heat waves, flooding, landslides, hurricanes or tropical storms.

#### 2.2.1 Eating-Related Eco-Concern Questionnaire

The Eating-Related Eco-Concern Questionnaire (EREC; Qi et al., 2022) is a 10-item self-report measure assessing the degree to which individuals worry about their food choices and their impact on climate change, (e.g., “I avoid eating any animal products due to my concerns about climate change”). Items were based on the authors’ clinical observations and the Climate Change Worry Scale (Stewart, 2021), as well as a literature review on eco-friendly eating and sustainable eating. Items are rated on a 5-point scale (1 = “Never” to 5 = “Always”). A total score is calculated as the sum of the items, with higher scores indicating greater eating-related eco-concern. The Italian version was developed using the back-translation method (Behling and Law, 2000). Two researchers translated the scale reaching a consensus and a bilingual individual then back-translated it into English to ensure linguistic equivalence. Refer to the Supplementary material for the finalized EREC Italian Version.

#### 2.2.2 Climate Change Worry Scale

The Climate Change Worry Scale (CCWS; Stewart, 2021) is a 10-item measure of worry about climate change (e.g., “Thoughts about climate change cause me to have worries about what the future may hold”). Items are rated on a 5-point scale (1 = “Never” to 5 = “Always”). The total score is computed as the sum of all items, with higher scores indicating greater climate worry. The scale demonstrated a one-factor structure, invariant across gender, with good internal consistency (*α* = 0.95) and test–retest reliability (*r* = 0.91) in the original study (Stewart, 2021). The validated Italian version used in this study (Innocenti et al., 2022) also showed validity and reliability. In the present study, internal consistency was *α* = 0.92.

#### 2.2.3 Eating Disorder Examination-Questionnaire

The Eating Disorder Examination-Questionnaire (EDE-Q; Fairburn, 1994) is a 28-item self-report questionnaire assessing disordered eating over the last 28 days. It contains four subscales: dietary restraint, with 5 items concerning restraint over eating, avoidance of eating, and dietary avoidance (e.g., “Have you been deliberately trying to limit the amount of food you eat to influence your shape or weight, whether or not you have succeeded?”); eating concern, with 5 items on preoccupation with food, eating in secret, and guilt about eating (e.g., “Has thinking about food, eating or calories made it very difficult to concentrate on things you are interested in, for example, working, following a conversation, or reading”); shape concern, with 8 items on the desire for a flat stomach, the importance of body shape, and fear of weight gain (e.g., “Have you had a definite desire to have a totally flat stomach?”); and weight concern, with 5 items on the importance of weight, dissatisfaction with weight, and the desire to lose weight (e.g., “Has your weight influenced how you think about (judge) yourself as a person?”). The remaining items concern frequency data of key behavioral features of EDs. Responses are on a 7-point scale (0–6), with subscale scores being the mean of subscale items, and higher scores indicating greater disordered eating. A global score is the mean of the four subscale scores. The EDE-Q has consistently shown good reliability and validity (Luce and Crowther, 1999; Bardone-Cone and Boyd, 2007; Mond et al., 2004, 2008; Aardoom et al., 2012). In this study, the Italian version (Calugi et al., 2017) was used, with Cronbach’s *α*s of 0.95 for the global score, 0.85 for restraint, 0.82 for eating concern, 0.92 for shape concern, and 0.84 for weight concern.

#### 2.2.4 Eating Habits Questionnaire

The Eating Habits Questionnaire (EHQ-21; Gleaves et al., 2013) is a 21-item self-report tool designed to gauge thoughts, actions, and emotions associated with an intense emphasis on healthy eating. It includes three subscales: knowledge of healthy eating, with 5 items regarding diet superiority (e.g., “I know more about healthy eating than other people”); problems associated with healthy eating, with 12 items on avoidance and social difficulties (e.g., “My healthy eating is a significant source of stress in my relationships”); and feeling positively about healthy eating, with 4 items (e.g., “I feel in control when I eat healthily”). Items are rated on a 4-point scale (1 = “false, not at all true” to 4 = “very true”). Higher scores indicate tendencies toward orthorexia nervosa. The Italian adaptation of EHQ-21 has shown to be valid and reliable in a general population sample (Novara et al., 2017). In this study, Cronbach’s *α* was 0.89 for the total score, 0.82 for knowledge, 0.85 for problems, and 0.62 for feelings.

### 2.3 Statistical analyses

To collect evidence of validity based on internal structure and evaluate whether the original one-factor model of the EREC is confirmed in the Italian context, a confirmatory factor analysis (CFA) using the weighted least square mean and variance adjusted (WLSMV) estimator, which is recommended for ordinal data (Li, 2016a, 2016b; Rhemtulla et al., 2012), was applied. Model fit was deemed acceptable if the root mean square error of approximation (RMSEA) and the standardized root mean square residual (SRMR) were ≤0.08, and the comparative fit index (CFI) and the Tucker–Lewis index (TLI) were ≥0.90 (Hu and Bentler, 1999). In case of poor fit, modification indices and the expected parameter change were examined to pinpoint areas of misfit and re-specify the model (Sellbom and Tellegen, 2019).

Reliability was assessed using ordinal *α* and ordinal *ω*, with values ≥0.70 considered acceptable (Nunnally, 1978; McDonald, 1999). Evidence of validity based on relations with other variables was gathered by computing Pearson’s correlations between EREC and CCWS, EDE-Q, EHQ-21, age, and BMI, and by testing differences in EREC scores between groups based on diet motivations (i.e., pro-environmental and/or ethical reasons vs. other reasons) and personal experience of climate change events (i.e., yes vs. no) using between-subjects ANOVA. Participants’ scores in EREC and CCWS were compared using repeated measures ANOVA.

The sample size was calculated beforehand to ensure a minimum of 10 observations for each parameter estimated in the CFA model (Kline, 1998). With 50 estimated parameters in the original one-factor model of the EREC, a minimum sample size of 500 was considered adequate. The threshold for statistical significance was set at *p* < 0.05. Interpretation of effect size was based on Pearson’s *r* (0.10 = small, 0.30 = medium, 0.50 = large) and Cohen’s *d* (0.20 = small, 0.50 = medium, 0.80 large) (Cohen, 1988). Analyses were conducted in R (version 4.3.3, https://www.r-project.org/), using the lavaan (Rosseel, 2012) and psych (Revelle, 2017) packages.

## 3 Results

### 3.1 Participant characteristics

The sample included 663 adults aged 18–73 years (*M* = 37.18, *SD* = 12.11). Most of the sample self-identified as female (85.1%) and of Italian nationality (97.9%), and 43.5% had a university degree or higher. Over two-thirds of participants were employed, and more than half (56.4%) reported no difficulty in currently making ends meet. Mean BMI for those who reported height and/or weight (*n* = 655) was 25.41 kg/m^2^ (*SD* = 6.05) for females and 25.43 (*SD* = 4.64) for males. As for dietary habits, most participants (81%) reported being omnivore, and the most frequently reported motivation for current dietary choices was taste preferences (62.9%). About one-third of the participants reported past personal experiences of extreme climate change events. See Table 1 for participant characteristics.

**Table 1:** Participant characteristics (*n* = 663).

	*n*	*%*
Gender		
Female	564	85.1
Male	87	13.1
Non-binary/Prefer to not respond	12	1.8
Nationality		
Italian	649	97.9
Other	14	2.1
Highest educational level		
Middle School	56	8.4
High school	283	42.7
Undergraduate degree	137	20.7
Postgraduate degree	151	22.8
Other	36	5.4
Currently employed		
Yes	451	68.0
No	212	32.0
Current income		
Some difficulties getting by	195	29.4
No difficulties getting by	217	32.7
Have to only worry about extra expenses	157	23.7
Great difficulties in getting by	41	6.2
Prefer not to respond	47	7.1
Self-classified dietary habits		
Omnivore	537	81
Flexitarian	39	5.9
Vegetarian	32	4.9
Pescetarian	33	4.8
Plant-based/Vegan	22	3.3
Motivations for dietary choices		
Health reasons	233	35.1
Weight management	158	23.8
Ethical or moral reasons	127	19
Environmental concerns	96	14.5
Taste preferences	417	62.9
Medical reasons	48	7.2
Religious or spiritual values	13	2
Past experience with extreme climate events		
Yes	240	36.2
No	423	63.8

### 3.2 Factor structure

**Figure 1:**
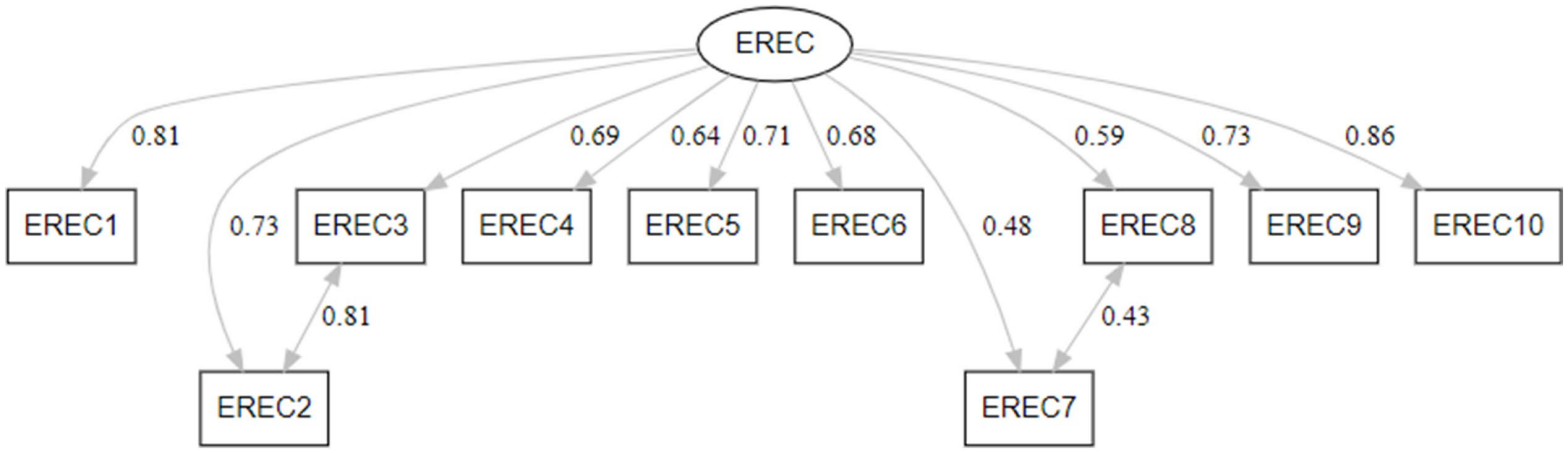
Eating-Related Eco-Concern (EREC) measurement model with standardized parameters.

The hypothesized one-factor model did not show an acceptable fit, *χ^2^*(35) = 587.053, *p* < 0.001, RMSEA = 0.154, SRMR = 0.113, CFI = 0.972, TLI = 0.964. Modification indices indicated that estimating the residual correlation between item #2 and item #3 would reduce the *χ^2^* by 317.177, with an expected parameter change of 0.478. Similarly, estimating the correlation between the residuals of item #7 and item #8 would decrease the *χ^2^* by 133.583, with an expected parameter change of 0.334. Thus, the model to include correlated residuals for these two pairs of items was re-specified. The re-specified model showed acceptable fit across all fit indices, *χ^2^*(33) = 162.793, *p* < 0.001, RMSEA = 0.077, SRMR = 0.062, CFI = 0.993, TLI = 0.991. Standardized factor loadings ranged from 0.48 to 0.87 (*p* < 0.001) (Figure 1).

### 3.3 Internal consistency

Internal consistency estimates were adequate, with *α* = 0.90, *ω* = 0.93, and an average item correlation of *r* = 0.48.

### 3.4 Relations with other variables

As shown in Table 2, EREC scores correlated significantly and positively with CCWS and EHQ-21 subscale and total scores, with strong and weak-to-moderate effect sizes, respectively. Among the EHQ-21 subscales, the strongest correlation was with the Knowledge subscale. The correlations with EDE-Q, age, and BMI were non-significant.

**Table 2:** Correlations with other variables and descriptive statistics.

	*r*	*M* (*SD*)	Range
EREC	–	26.32 (8.21)	10–47
CCWS	0.57^*^	29.80 (8.48)	10–49
EDE-Q-Restraint	0.01	2.05 (1.82)	0–6
EDE-Q-Eating concern	−0.03	1.26 (1.45)	0–6
EDE-Q-Shape concern	−0.08	2.78 (1.92)	0–6
EDE-Q-Weight concern	−0.07	2.35 (1.80)	0–6
EDE-Q-Total	−0.05	2.10 (1.54)	0–5.95
EHQ-Knowledge	0.42^*^	12.50 (3.47)	5–20
EHQ-Problems	0.28^*^	19.34 (6.24)	12–45
EHQ-Feelings	0.23^*^	10.42 (2.75)	4–16
EHQ-Total	0.36^*^	42.26 (10.61)	21–81
Age	0.07	37.18 (12.11)	18–73
BMI	−0.07	25.40 (5.91)	15.43–49.31

*p ≤ 0.001.

Participants who cited pro-environmental and/or ethical reasons among their motivations for dietary choices (*n* = 154, *M* = 32.08, *SD* = 7.70) reported significantly higher EREC scores than participants who listed other reasons (personal health, weight control, religious or spiritual values, and taste preferences) (*n* = 509, *M* = 24.58, *SD* = 7.54), with a large effect size [*F*(1,661) = 116.03, *p* < 0.001, *d* = 0.99]. Participants who reported having experienced extreme climate change events (*n* = 240, *M* = 27.61, *SD* = 8.26) showed significantly, slightly higher EREC scores than participants with no such experience (*n* = 423, *M* = 25.59, *SD* = 8.10) [*F*(1,661) = 9.38, *p* = 0.002, *d* = 0.25]. Participants scored significantly higher in CCWS than in EREC, with a small-to-medium effect size [*F*(1,662) = 133.22, *p* < 0.001, *d* = 0.42].

## 4 Discussion

This study investigated the psychometric properties of an Italian translation of the EREC, a recently developed screening tool designed to assess eating-related concerns and behaviors related to climate change. Altogether, the Italian EREC has shown evidence of both validity and reliability. As evidence of validity based on internal structure, the results from CFA indicated that the original one-factor model fit the data well, after adding error covariation between items #2 (“I avoid eating meat due to concerns about climate change”) and #3 (“I avoid eating any animal products due to my concerns about climate change”), and between items #7 (“I avoid genetically modified foods due to concerns about biodiversity loss”) and #8 (“I try to only eat organic foods or food produced without pesticides”). Although these covariations were not reported in the original EREC validation study, they appear logically and theoretically justified, as it is plausible to assume that the error terms of these items share some variance. Indeed, items #2 and #3 both target dietary modifications motivated by the same environmental concern, more precisely the impact of meat and animal product consumption on climate change. This is in line with prior research reporting a robust link between environmental concern and the act of avoiding specific types of foods, especially meat and all animal products (Sanchez-Sabate and Sabaté, 2019; Turnes et al., 2023). As for items #7 and #8, both focus on food consumption choices aimed at preserving environmental integrity. Coherently, a link between ecological responsibility and personal health concerns has been reported in several studies (Cheah et al., 2020; Lai et al., 2020; Turnes et al., 2023).

To further support the unidimensionality of the EREC scale, internal consistency estimates were high, and very close in value to those reported in previous psychometric studies (Qi et al., 2022; El Zouki et al., 2024). Regarding the expected relations with other variables, as in the American validation sample, EREC had a positive, large correlation with the CCWS (Stewart, 2021). Consistent with the original study, the mean score in EREC was lower than in CCWS, indicating that those who are concerned about climate change do not necessarily alter their eating behaviors accordingly (Qi et al., 2022). Indeed, the relationship between the similar construct of eco-anxiety and dietary behaviors is complex, with eco-anxiety correlating significantly with some aspects of healthy and sustainable eating but not with others (Kabasakal-Cetin, 2023). EREC scores were unrelated to younger age, despite similar constructs like climate anxiety and eco-anxiety often being found to affect younger individuals more than older counterparts (Clayton and Karazsia, 2020; Rocchi et al., 2023). This lack of association suggests that, in the Italian context, climate-related concerns may not be exclusive to younger generations. One possible explanation is the increasing frequency and intensity of climate change events, such as extreme heat waves (Spano et al., 2021), which disproportionately impact older adults due to their heightened health vulnerabilities. As a result, eco-concern may extend beyond young adults to include older individuals who are directly affected by these events. Contrary to hypotheses, none of the EDE-Q subscales nor BMI correlated significantly with the EREC. However, these results highlight the lack of overlap between eating-related eco-concern and EDs, as also noted in the original validation study.

As expected, based on the literature, orthorexia nervosa symptomatology correlated positively with EREC scores, with a medium effect size. Concerns about one’s food choices and the impact on climate change might lead some individuals to enact rigid eating patterns that parallel orthorexia nervosa behaviors. Indeed, a recent review suggests that following a vegetarian diet, which is increasingly chosen due to ecological and sustainability concerns (Mayrhofer et al., 2024), is associated with orthorexic eating behaviors (Brytek-Matera, 2021). EREC scores correlated more strongly with the Knowledge subscale, which contains items regarding the superiority of one’s diet compared to others, than with the other EHQ-21 subscales. This suggests that worrying about one’s food choices due to its possible negative impact on climate change, captured by the EREC, may be accompanied by or motivated in part by feelings of moral superiority to others, given the ethical implications of one’s dietary choices (Fox and Ward, 2008) and the moral values associated with more climate-friendly diets and avoidance of animal products (Rozin et al., 1997; Ioannidou et al., 2023).

Finally, as further evidence of validity based on relations with other variables, individuals who adopted their diet for pro-environmental and/or ethical reasons reported considerably higher EREC scores compared to individuals with other dietary reasons. Indeed, interest in environmental sustainability is associated with eco-anxiety (Chung et al., 2023), and well-known associations exist between climate-related concerns and pro-environmental behaviors (Verplanken et al., 2020; Jain and Jain, 2022; Heeren et al., 2023; Ogunbode et al., 2022). Additionally, individuals with personal experiences of climate change events showed slightly higher EREC scores than those without these occurrences. This aligns with the well-documented relationship between experiencing events associated with climate change and increased worries and negative emotions about climate change itself and climate-related issues (Bergquist et al., 2019; Clayton, 2020; Sambrook et al., 2021). While the current study only collected information on exposure to climate-related events, the number and severity of such events each participant had encountered could have added valuable insight and should be considered in future research.

This study has certain limitations that should be considered. The use of social media as the major recruitment channel in this study may have influenced the characteristics of the sample, potentially attracting individuals who are more active in online environmental discussions or who are more exposed to digital campaigns about climate change, potentially affecting the representation of environmental concerns in this study. Additionally, this recruitment approach may limit generalizability, as individuals who are less engaged in online spaces or environmental conversations may have different levels of climate-related eating concerns that are underrepresented here. The sample was highly unbalanced in terms of gender, with 85% of participants self-identifying as female. Consequently, gender comparisons were not conducted in this study. Interestingly, the original validation study found no significant gender differences in EREC scores, even though broader research suggests an association between female gender and greater climate-related concerns (Qi et al., 2022). While this might indicate that gender has limited influence on EREC scores, future studies with more balanced samples should investigate this further to confirm whether gender differences are truly absent in the context of eating-related eco-concerns. Finally, test–retest reliability and sensitivity to change were not assessed, both of which should be included in future psychometric evaluations of the Italian EREC using a longitudinal design to establish its stability over time and its responsiveness to shifts in climate-related behaviors or attitudes.

### 4.1 Clinical implications

The clinical implications of the present study’s findings are multifaceted. Firstly, the successful adaptation of the Italian EREC underscores its potential utility in assessing eating-related concerns and behaviors related to climate change with a brief and time-efficient measure. The evidence of validity and reliability of the scale suggests its viability for both research and clinical settings, including mental health services, as well as for use by nutritionists and dietary specialists, given its ability to assess concerns and behaviors related to dietary choices and eating habits. This tool may also serve as a valuable resource in public health initiatives focused on promoting sustainable eating behaviors. Indeed, the observed correlations between EREC scores and variables like climate change worry and orthorexia nervosa symptomatology highlight the complex interplay between climate change, climate concerns, and mental health (Clayton, 2020; Cianconi et al., 2023). Notably, these findings introduce the additional domain of eating behaviors as an understudied topic within the climate change context (Rodgers et al., 2023), adding further dimensions to the ongoing discourse on eco-anxiety and climate-related health impacts. Future studies are necessary to further elucidate the intricate relationships between climate change concerns and dietary behaviors spanning from sustainable and functional to dysfunctional, disordered behaviors. Specifically, longitudinal research could better establish the causal relationships among eco-concerns, dietary behaviors, and disordered eating, shedding light on how these dynamics evolve over time.

In the clinical context, these findings underscore the importance of considering climate-related factors in the assessment and treatment of EDs and disordered eating, particularly orthorexia nervosa. Moreover, clinicians should be attentive to the moral dimension of dietary choices in clinical practice, as indicated by the association between EREC scores and feelings of moral superiority, an important aspect of orthorexia nervosa (Cheshire et al., 2020). Acknowledging these moral aspects in patient care may enhance treatment outcomes by addressing underlying motivations for restrictive eating patterns. Additionally, the observed associations underscore the ever-growing importance of addressing eco-anxiety and eco-concerns in both prevention efforts and mental health promotion. Despite the increasing relevance of eco-concerns and eco-anxiety in clinical practice, clinicians lack information on how to manage such feelings in therapeutic contexts (Trost et al., 2024). Integrating training in eco-anxiety and eco-concerns in clinician education could help bridge this gap, equipping professionals with the skills needed to support individuals experiencing heightened climate-related stress.

## Data Availability

The raw data supporting the conclusions of this article will be made available by the authors, without undue reservation.
