# Supplementary material for: Validation of the Italian version of the Eating-Related Eco-Concern Questionnaire: insights into its relationship with orthorexia nervosa
Source: Front Psychol. 2024 Nov 27;15:1441561. doi: 10.3389/fpsyg.2024.1441561 (PMC11632223; doi:10.3389/fpsyg.2024.1441561)
Supplement: Supplementary file 1 [file Table_1.DOCX]

**Supplementary Material.**

Eating-Related Eco-concern Questionnaire (EREC)- Italian Version (Tecuta et al., 2024)

| 1. Passo più tempo delle altre persone a cercare cibo sostenibile. |
| --- |
| 1. Evito di mangiare carne a causa di preoccupazioni riguardanti il cambiamento climatico. |
| 1. Evito di mangiare qualsiasi prodotto animale a causa delle mie preoccupazioni riguardanti il cambiamento climatico. |
| 1. Cerco di non sprecare cibo a causa di preoccupazioni riguardanti il cambiamento climatico. |
| 1. Incoraggio attivamente gli altri a cambiare i loro comportamenti per rallentare il cambiamento climatico. |
| 1. Cerco di mangiare meno a causa delle mie preoccupazioni riguardanti il cambiamento climatico. |
| 1. Evito cibi geneticamente modificati a causa di preoccupazioni riguardanti la perdita di biodiversità. |
| 1. Cerco di mangiare solo cibi biologici o cibi prodotti senza pesticidi. |
| 1. Evito cibi venduti in imballaggi eccessivi o non riciclabili. |
| 1. Presto molta attenzione alle informazioni riguardanti l’impatto che certi cibi hanno sull’ambiente (es. pesca eccessiva, gas serra, irrigazione). |
